# Pursuing Experimental Reproducibility: An Efficient Protocol for the Preparation of Cerebrospinal Fluid Samples for NMR-Based Metabolomics and Analysis of Sample Degradation

**DOI:** 10.3390/metabo10060251

**Published:** 2020-06-16

**Authors:** Benjamin Albrecht, Elena Voronina, Carola Schipke, Oliver Peters, Maria Kristina Parr, M. Dolores Díaz-Hernández, Nils E. Schlörer

**Affiliations:** 1 Department of Chemistry, Universität zu Köln, Greinstr.4, 50939 Köln, Germany; b.albrecht@uni-koeln.de (B.A.); elena.voronina@uni-koeln.de (E.V.); 2 Charité– Universitätsmedizin Berlin, Corporate Member of Freie Universität Berlin, Humboldt-Universität zu Berlin, and Berlin Institute of Health, Experimental & Clinical Research Center (ECRC), Lindenberger Weg 80, 13125 Berlin, Germany; schipke@predemtecdx.com; 3 Department of Psychiatry and Psychotherapy, Charité-Universitätsmedizin Berlin, Campus Benjamin Franklin, Hindenburgdamm 30, 12203 Berlin, Germany; oliver.peters@charite.de; 4 Institute of Pharmacy, Freie Universität Berlin, Königin-Luise-Str. 2-4, 14195 Berlin, Germany; maria.parr@fu-berlin.de

**Keywords:** cerebrospinal fluid (CSF), NMR metabolomics, sample preparation, pH, sample degradation, time and temperature dependence, standard operating procedures (SOP)

## Abstract

NMR-based metabolomics investigations of human biofluids offer great potential to uncover new biomarkers. In contrast to protocols for sample collection and biobanking, procedures for sample preparation prior to NMR measurements are still heterogeneous, thus compromising the comparability of the resulting data. Herein, we present results of an investigation of the handling of cerebrospinal fluid (CSF) samples for NMR metabolomics research. Origins of commonly observed problems when conducting NMR experiments on this type of sample are addressed, and suitable experimental conditions in terms of sample preparation and pH control are discussed. Sample stability was assessed by monitoring the degradation of CSF samples by NMR, hereby identifying metabolite candidates, which are potentially affected by sample storage. A protocol was devised yielding consistent spectroscopic data as well as achieving overall sample stability for robust analysis. We present easy to adopt standard operating procedures with the aim to establish a shared sample handling strategy that facilitates and promotes inter-laboratory comparison, and the analysis of sample degradation provides new insights into sample stability.

## 1. Introduction

Cerebrospinal fluid (CSF), a secretion product of the central nervous system (CNS), is in dynamic exchange with the nerve tissue and circulates metabolites. Its composition indirectly reflects the biochemical processes occurring in the brain [1]. More recently, CSF metabolomics has received much attention in the research of neurological disorders like Alzheimer’s, Parkinson’s disease, multiple sclerosis or brain injury, amongst others [2,3,4,5,6,7,8].

NMR spectroscopic analysis of CSF in the field of metabolomics has gained importance due to the minimal requirement for sample preparation, and its ability to simultaneously detect and quantify a wide range of compounds. A fairly simple composition, low lipid and protein content [9,10,11]—compared to biofluids like blood or urine—make CSF an ideal candidate for NMR analysis, and indeed, its feasibility was demonstrated some thirty years ago [12]. Yet, handling of CSF samples still remains challenging [13] and some practical issues related to sampling procedure and sample stability, particularly pH control [14], require special attention.

Standardized collection of CSF and biobanking protocols have been established benefitting from clinical environments [15,16,17], but study-specific CSF sample preparation procedures at different research facilities prior to NMR spectroscopic analysis vary in key steps of the procedures. This is a critical aspect because variance introduced by sample preparation needs to be kept at a minimum to allow for comparability of results: NMR spectral features such as a signal-to-noise ratio, metabolite peak position (resonance frequency) and line shapes are affected by physiochemical parameters, thereby impacting compound identification and quantification accuracy.

The reported procedures in the literature differ in sample handling (e.g., storage duration/temperature), native sample volume, constitution (e.g., lyophilized samples) and final composition (e.g., sample/buffer-ratio). Remarkably, pre-analytical factors such as sample pH and ionic strength, native sample dilution, and treatment of high-molecular weight compounds differed amongst the consulted research articles, suggesting the lack of a common and comprehensive strategy, although it would be highly desirable.

We hereby propose a standard operating procedure designed to mitigate the influences of the aforementioned pre-analytical factors and present an efficient and simplified protocol for CSF sample preparation for NMR analysis. The presented protocol has been evaluated by analyzing authentic human CSF samples under the aspect of pH and (thereby) proton chemical shift consistency, and overall sample stability. Degradation was monitored in a two-sided scheme representing routine NMR laboratory conditions (i.e., experimental temperature and acquisition/waiting time).

## 2. Results and Discussion

A careful review of sample preparation procedures for the analysis of CSF samples by NMR spectroscopy described in the literature revealed that a large variety of protocols were adopted in the last fifteen years (Table 1) [4,5,6,7,8,13,18,19,20,21,22,23,24,25,26,27,28,29,30,31,32,33,34,35,36,37,38,39]. We have investigated the reliability of a self-designed protocol for the preparation of CSF samples, which enables the acquisition of NMR data compatible with small molecules spectra libraries used for NMR metabolomic profiling, thus preventing data misinterpretation.

Since CSF sample stability as well as interpretability of spectral data (i.e., chemical shift values) depend mainly on pH and, to a lesser extent, on osmolality and ionic strength, we have mainly focused on different aspects of pH control of CSF samples. In contrast to the substantial buffer capacity existing in blood/plasma, for example, the only acid-base homeostatic mechanism in CSF is a (native) bicarbonate buffer system. Immediately after sampling and exposure to an atmosphere with a non-physiological level of CO_2_, CSF is highly prone to undergo changes in pH [40,41]. A rising pH is the consequence, leading to decreased stability of metabolites sensitive to pH changes and, in addition, chemical shift changes that impede data analysis. In the reviewed literature, we did not find general agreement about protocol (experimental conditions), or optimal pH values for NMR-based metabolomics studies of CSF. Some studies did not report or adjust sample pH [8,23,24,26,32,35], while others manually adjusted it by adding acid/base [4,7,19,28,29,35,37] or employed a phosphate buffer with varying buffer capacity [5,6,13,18,20,21,25,27,31,33,34,38,39].

**Figure 1:**
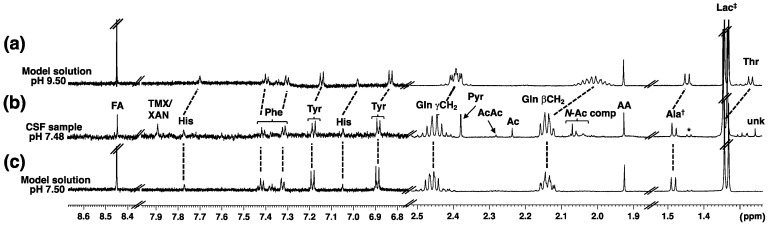
Partial one-dimensional ^1^H NMR spectra of the model solution (**a**,**c**) and an authentic CSF sample (**b**). The impact of a rise in pH can be clearly seen following the resonance positions of His, Phe, Tyr, Gln, Ala and Thr. TMX/XAN = xanthine/caffeine (tentative assignments, see Supplementary Materials for discussion), Ala^†^ = Alanine + unknown, * = lactate ^13^C satellite, Lac^‡^ = lactate + Thr, unk = unknown. Note: For compound abbreviations see Table 2.

We prepared first a model solution containing 13 common CSF metabolites (see Materials and Methods, and Figure S1, Supplementary Materials) and investigated aspects of pH control and buffer performance. Spectra of the model solution at different pH values were recorded to explore the influence of pH on spectral properties of CSF metabolites. In this way, an optimal pH for work with authentic CSF samples was defined. When taking the specificities of CSF into consideration, employing a buffer offers the only sustained pH control. Different literature-reported phosphate buffer ratios and compositions were tested using the model solution. A phosphate buffer system H_2_PO_4_^−^/HPO_4_^2−^ (pK_a_ 6.8) with an ideal buffer capacity in the pH range of 5.8–7.8 is well suited for pH control in CSF samples—native human CSF has a pH of 7.3. Adjusting buffer pH to a slightly lower value, pH 7.2, compensates for inter-sample pH variability and keeps the pH of samples in the range of native CSF [33,48]. In Figure 1 the 1D ^1^H NMR spectra of a CSF sample at pH 7.48 along with two spectra of the model solution at different pH values are shown demonstrating the impact of a pH increase (e.g., as a result of improper handling of a native CSF sample).

Initially we used, as previously reported in the literature [49,50], an internal pH indicator (i.e., imidazole) to monitor sample pH, thus avoiding individual measurements with a glass pH electrode, and also preventing potential sample contamination in this process. However, the results were not as accurate as expected, and the potential of overlapping signals of imidazole with a number of metabolite signatures in the aromatic region (e.g., if added with the buffer) led us to abandon this strategy. Instead, we measured the pH of the NMR samples after recording of spectra with a pH electrode for NMR tubes (Supplementary Materials Figure S2) to monitor buffer performance.

Furthermore, cation binding/complexation and ionic strength were identified to be important. A non-negligible correlation between chemical shifts, as well as peak shapes of some metabolites (e.g., citrate, glutamine and glucose) and osmolality was reported in an earlier study [48]. The buffer solution selected by us, consisting of mixed sodium and potassium phosphate salts, accounts for total sodium and potassium concentration without (additional) need to add corresponding chloride salts. An increased buffer volume accommodates varying concentration ranges of ionic species, e.g., lowering overall concentration of Ca^2+^ in solution while avoiding an excess of Na^+^, both known to affect peak shapes and chemical shifts [51,52].

In agreement with a previous study [49], our findings suggest that there is no need for protein removal to eliminate a broad signal underground (i.e., signals from macromolecular compounds), either by centrifugation, filtration or precipitation. Sample concentration by lyophilization should also be avoided if possible. It possibly only offers benefits for small sample volumes, is time consuming and prone to introducing quantitation uncertainties (e.g., side reactions [3] and loss of volatile metabolites [3,53]).

For metabolite identification and quantitation, a reference compound at a known concentration, either DSS (4,4-dimethyl-4-silapentane-1-sulfonic acid) or TSP (sodium 3-trimethylsilylpropanoate), is commonly added. DSS is known to be less affected by low pH values and higher protein concentrations [54], both not common in CSF. Therefore, we recommend the use of TSP-*d*_4_, which is significantly less costly. Possibly out of cost restraints some studies used non-deuterated DSS [49,50]. Such practice should be discouraged because of resulting peak overlap in the chemical shift region of aliphatic compounds hampering identification and quantification.

**Figure 2:**
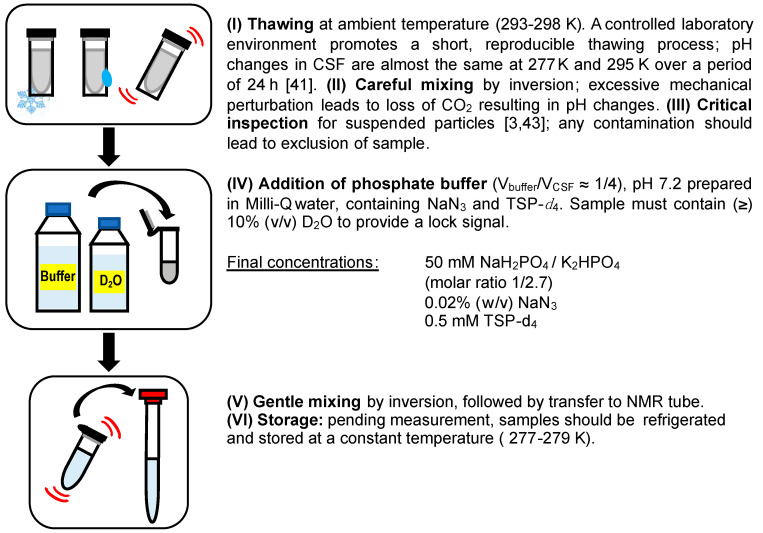
Proposed CSF sample preparation workflow detailing key aspects of sample handling and pH control (buffer composition described in step IV).

Taking all these aspects into consideration, we envisioned a protocol for the preparation of CSF samples, which was further validated. An illustration of the whole procedure is shown in Figure 2.

**Figure 3:**
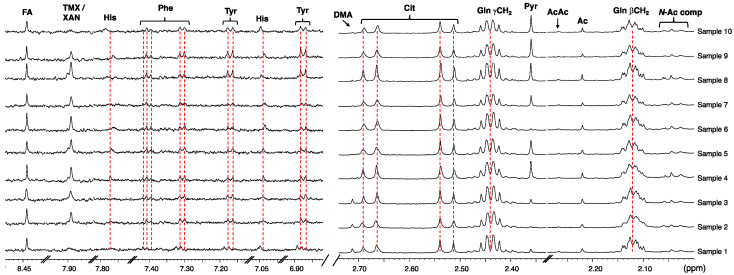
Stack plot of two spectral regions of the proton spectra of 10 CSF samples prepared as described in text showing consistency of metabolite resonances typically affected by differences in ionic strength and pH (600 MHz, 298 K, phosphate buffer 50 mM, pH 7.30–7.69, 10% D_2_O, line broadening = 0.5 Hz). Note: For compound abbreviations, see Table 2.

We carried out an NMR study on a small cohort of CSF samples (*n* = 23) and observed an excellent buffer performance, with a reproducible effect on chemical shift/peak shape. The measured pH after addition of buffer was 7.52 ± 0.16 (range = 7.36–7.68). Excerpts of ^1^H NMR spectra of ten representative CSF samples are shown in Figure 3 demonstrating excellent alignment of chemical shifts of metabolites known to be (strongly) dependent on sample pH. Leveling alterations in the ionization equilibria of metabolites induced by pH and salinity leads to the observed chemical shift consistency of metabolite peaks. Following data acquisition, we monitored the pH of several samples (stored at 277 K) over three days and did not observe significant pH changes.

The quality of the recorded spectra from samples of this small cohort, prepared according to the experimental protocol described above, allowed for the identification of thirty-eight metabolites (MSI, level 2) [55] across all spectra. The concentration estimates of metabolites of one of the pooled samples (see below) derived from peak integration are given in Table 2.

In some of the 23 samples, possible non-endogenous contaminants (i.e., ethanol and/or isopropanol) were identified and we determined their concentration in a few cases (Supplementary Materials Figure S3). Their presence seems to be linked to sample collection and sterilization during sample collection at hospital sites. Detection of ethanol in CSF might be of endogenous and/or exogenous origin [56], but our findings suggest an exogenous origin, even though we could not rule out lower levels of ethanol being masked by the application of skin disinfectant. In some samples we also noticed the presence of caffeine/xanthine (Table 2). To our knowledge, the detection of caffeine by ^1^H NMR was previously not reported in studies analyzing human CSF, though it has been detected by LC/MS [57]. We identified peaks with chemical shifts that match those of caffeine (δ = 7.891, 3.944, 3.519 and 3.339 ppm) when compared to literature values (Supplementary Materials, Figure S4). Spectral profiling yielded a concentration range of 2.3–15.6 µM. The assignment was marked as ‘tentative’ because xanthine only had one peak with a chemical shift very similar to the one of caffeine; in cases where the peak at δ = 7.891 ppm displayed larger line widths, it is likely that both compounds were present.

Our findings described above, shed light on possible sources of contaminants and underscore the importance of accurate control/standardization of pre-analytical factors (e.g., sample collection, storage and sample preparation) for yielding reliable data for statistical evaluation. With this in mind, we were interested to know whether an inherently quantitative NMR analysis could provide insights to unravel the processes causing sample decay, and thereby identify potential markers of CSF degradation.

Two pooled CSF samples, prepared following the proposed procedure, were used to examine sample temperature stability and their degradation as a function of time. Both samples were pooled from remainders of different aliquots of unique CSF samples. One sample was stored at 277 K (‘LT’ = low temperature sample), the other one at 294 K (‘RT’ = room temperature sample) and both were monitored for several days. Sample pH was determined, and spectra of each sample were acquired at different points in time. The different temperatures address two common scenarios in NMR-based metabolomics studies: (1) storage of samples while queued for acquisition (i.e., in a cooling rack), and/or (2) during possible delays at room temperature prior to measurement. Jointly, this allowed the analysis of degradation pathways for certain metabolite classes.

In the ‘LT’ sample, the pH measured after sample preparation was 7.30 and it remained unchanged after 2.5 h. Moreover, at t = 96 h it was only slightly higher (i.e., pH = 7.56). The time evolution of metabolites concentrations of the ‘LT’ sample shows little variance within the first 48 h of storage (Supplementary Materials, Table S1). While stored for this time span at 277 K, acquisition time at room temperature totaled 150 min. Spectral features remained almost unchanged (Supplementary Materials, Figure S5). During the observation period of 48 h relative metabolite concentrations of most profiled metabolites (i.e., 89% of the total number of metabolites) deviated by less than 8.5%. Only four metabolites (i.e., acetoacetate, dimethylamine, histidine, TMAO) present in the low concentration range (c < 7 µM) had larger deviations of 10–16% (that are likely the result of quantification uncertainty at low concentration).

As expected, the non-refrigerated storage of the ‘RT’ sample led to larger compositional changes caused by degradation in a contrastable storage interval of 52 h at constant temperature. Percent metabolite concentration changes range from increased levels in acetate (531%), pyroglutamate (113%) or dimethylamine (71%) to decreased levels in ascorbate (100%), pyruvate (86%) or fructose (21%; Supplementary Materials, Table S2). Prolonged storage of ‘RT’ sample (i.e., exceeding 52 h) is accompanied by further changes. Two metabolites could not be detected after storage (ascorbate and hypoxanthine), and other metabolite levels already decreasing at 52 h continued to fall (pyruvate, fructose and threonine) while most other amino acid derivatives (leucine, isoleucine, 2-hydroxyisovalerate, valine, 2-hydroxybutyrate and alanine) followed this trend after prolonged storage. Surprisingly, 14 metabolite levels (e.g., acetone, citrate, creatine, creatinine, dimethyl sulfone, formate, methanol, histidine and myo-inositol) remained almost unchanged during the 100-h long observation period.

**Figure 4:**
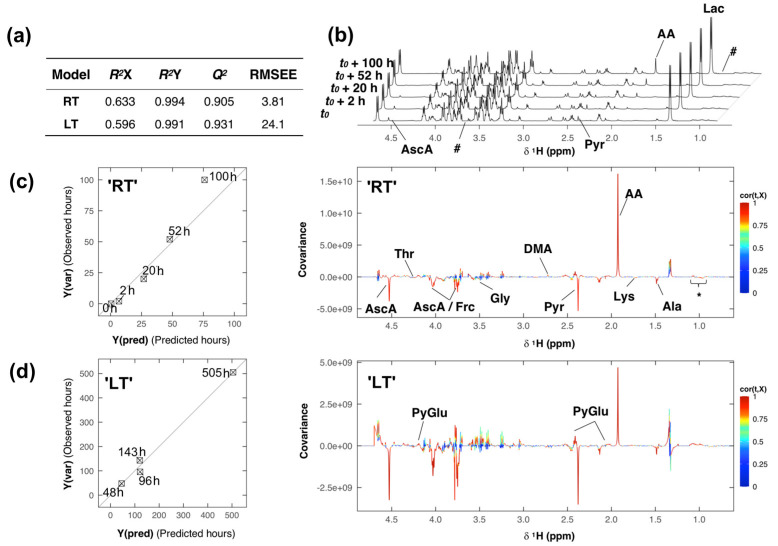
Sample degradation analysis of the room temperature (‘RT’) and low temperature (‘LT’) samples. (**a**) Table of partial-least squares (PLS) model performance and quality parameters. (**b**) ^1^H NMR spectra of the ‘RT’ sample acquired at different time intervals. (**c**) (left) Cross-validated predictions for sample ‘RT’, and (right) loading plot derived from the PLS model of sample ‘RT’. (**d**) (left) Cross validated predictions for sample ‘LT’, and (right) loading plot derived from PLS model of sample ‘LT’. Note: For compound abbreviations, see Table 2; here: * = VAL, LEU, aHBA and bHMB; # = regions where signals of ethanol/isopropanol were deleted.

To more accurately compare the entire degradation process in the two samples stored at different temperatures, the full-resolution spectral data was subjected to a partial-least squares (PLS) regression analysis. Two separate PLS models (Figure 4) were built by regressing the Pareto-scaled spectral data with respect to time (‘RT’ *t*(h) = 0, 2, 20, 52, 100; ‘LT’ *t*(h) = 48, 96, 143, 505).

Model complexity was optimized by leave-one-out cross-validation, and both models were computed with one PLS component only. The performance and quality parameters of the PLS models are presented in Figure 4a. The proportion of variance in the spectral data explained (indicating goodness of fit) by each model was about 60%, R^2^X = 0.633 (‘RT’) and 0.596 (‘LT’), and close to 100% for the response variable, R^2^Y = 0.994 (‘RT’) and 0.991 (‘LT’). Each model’s predicted variation Q^2^ (i.e., goodness of predictability) was high, 0.905 in the ‘RT’, and 0.931 in the ‘LT’ model. The cross-validated predictions Y(pred) versus measured Y-variables Y(var) for each model, ‘RT’ and ‘LT’ respectively, with root-mean-square errors of estimation (RMSEE) of 3.81 and 24.1 are shown on the left in Figure 4c,d; respectively and the diagonal in each plot represents the target line.

Spectral features that are correlated with the modeled changes occurring in each sample, can be visualized and identified in the back-transformed loading plot (Figure 4c,d (right)), showing the covariance cov(t,X) between the predictive score t and the centered X-variables (spectral data), colored according to the absolute value of the correlation loading cor(t,X). Spectral features that are correlated with the modeled changes occurring in each sample, can be visualized and identified in the back-transformed loading plot (Figure 4c,d (right)), showing the covariance cov(t,X) between the predictive score t and the centered X-variables (spectral data), colored according to the absolute value of the correlation loading cor(t,X). Features that show high absolute correlation loadings have high model influence. The covariance and correlation structure between predictive score and spectral data looks very similar in both models (Figure 4c,d (left)), apart from some derivative effects in the loading plot of the ‘LT’ sample, resulting from line shape artifacts and positional noise accompanying the storage over 21 days. Spectral features, i.e., metabolites, that have high model influence were identified to be, e.g., ascorbic acid, threonine, fructose, glycine, dimethylamine, pyruvate, pyroglutamate, acetate, lysine and alanine.

**Figure 5:**
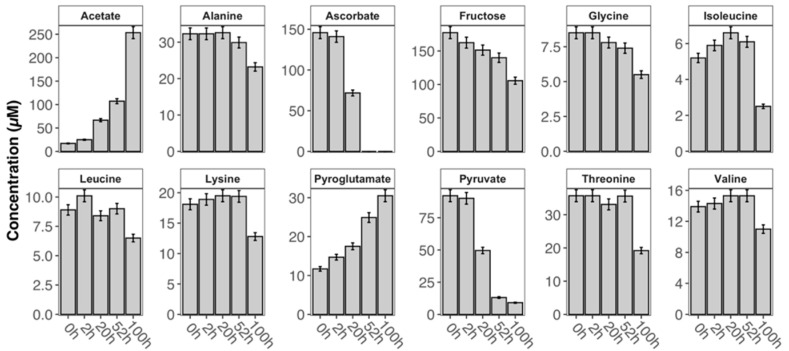
Time dependence of the relative concentration profiles of selected metabolites in the CSF sample ‘RT’ (error bars represent a 5% quantification uncertainty).

A group of 12 metabolites was selected, exhibiting high correlations and loadings in the predictive component of the PLS model of the ‘RT’ sample, indicating variance in the levels of those metabolites in the observed time interval. The relative concentration levels of those metabolites (Table S2) obtained at varying intervals are presented in Figure 5 and exhibited noticeable changes: most metabolite levels decreased because of decomposition with the exception of acetate and pyroglutamate whose levels increased.

The distinct changes in the metabolite levels of acetate and pyruvate, as well as ascorbate in the ‘RT’ sample (Figure 4b) were further investigated by statistical total correlation spectroscopy analysis (i.e., STOCSY, Supplementary Materials, Figure S6). Using the acetate peak (δ = 1.937 ppm) as a driver, very high correlations was observed with both pyruvate (r^2^ ≈ 1, δ = 2.379 ppm) and ascorbic acid (r^2^ ≈ 0.8, δ = 4.528, 4.035 and 3.787 ppm). Relative concentration changes Δc_PYR_~Δc_AA_ were highly correlated for a duration of 20 h, supporting a putative interconversion (Supplementary Materials, Table S3).

After twenty hours of storage at room temperature though, the relative concentration change of pyruvate was smaller than that of acetate (Figure 5). Upon consumption of pyruvate (*t* > 100 h), acetate concentration continued to rise, possibly due to bacterial activity not fully suppressed by sodium azide. Additionally, breakdown of lower concentrated *N*-acetylated compounds present in solution may also be responsible for releasing acetate. The correlation of this process (as picked up by the STOCSY analysis) to ascorbate can be explained by the shared linear dependency of the overall degradation (i.e., change in intensity).

A comparable relative concentration change of acetate and pyruvate was observed in the ‘LT’ sample after 21 days (505 h) of storage at 277 K, highlighting the commonality of this degradation pathway across these two pooled samples, which is also the result of the PLS model comparison.

While several metabolite classes only exhibited minor changes in their concentration levels in both samples in a moderate observation and storage period (48/52 h), at room temperature clear signs of active degradation are observable. Our findings strongly indicate to exercise caution when considering any of the affected metabolites (e.g., acetate, pyruvate and ascorbic acid) as markers. Particularly ascorbic acid is susceptible to oxidative degradation, especially at room temperature. In one study three metabolites were found to be significant in the differentiation of the studied groups [7], while another one excluded pyruvate and acetate, as well as acetone because of unspecified apparent batch effects [20]. The changes in acetate and pyruvate concentrations found in our experiments under both storage conditions may indicate potential ongoing enzymatic activity causing the interconversion that is part of the TCA (tricarboxylic acid) cycle. In a small cohort of CSF samples that were stored at low temperature for one month, the changes in the levels of acetate and pyruvate (Supplementary Materials, Table S4) were found to be statistically significant (Supplementary Materials, Figure S7), supporting metabolite interconversion, while the significant rise of acetate in the ‘RT’ sample is likely caused by the breakdown of low-concentrated macromolecules and of *N*-acetylated compounds. Observations supporting this causal relationship of ongoing aerobic and anaerobic metabolic processes were made elsewhere [42]. Further research is necessary to delineate the dependency of their decay. The uniform approach presented in this work may help to validate biomarker research in CSF samples.

## 3. Materials and Methods

### 3.1. Materials and Reagents

All compounds used for the model solution (sodium acetate, l-alanine, trisodium citrate, sodium formate, glucose, l-glutamine, sodium lactate, methanol, l-phenylalanine, l-histidine, l-tyrosine, l-threonine and l/d-valine) as well as sodium azide, sodium phosphate monobasic and potassium phosphate dibasic were purchased from Sigma-Aldrich and were used without further purification.

D_2_O and deuterated sodium 3-trimethylsilylpropionate (TSP-*d*_4_) used as a chemical shift reference were purchased from Cambridge Isotope Laboratories.

### 3.2. CSF Collection

The cerebrospinal fluid (CSF) samples analyzed in this study were obtained from a local biomaterial bank. The study was approved by an institutional review board (Ethikausschuss 2 Charité; BIH CRG 2a, EA2/118/15). Informed consent was obtained from all individuals prior to participating in this study. Aliquots of 0.5–1 mL CSF per subject were collected between 9 and 12 a.m. Patients did not fast prior to lumbar puncture. CSF was collected in sterile polypropylene vials and centrifuged immediately (10 min, 3000× *g* at RT). The supernatant was aliquoted in cryotubes, frozen in liquid nitrogen max. 30 min after lumbar puncture and subsequently stored at 193 K until ^1^H NMR analysis.

### 3.3. Preparation of Model Solution and CSF Samples for NMR Studies

#### 3.3.1. Model Solution

The stock solution was prepared in Milli-Q water and contained sodium acetate (0.108 mM, l-alanine (0.091 mM), trisodium citrate (0.307 mM), sodium formate (0.135 mM), glucose (2.50 mM), l-glutamine (0.599 mM), l-histidine (0.029 mM), sodium lactate (1.41 mM), methanol (0.247 mM), l-threonine (0.073 mM), l/d-valine (0.067 mM), l-tyrosine (0.033 mM) and l-phenylalanine (0.019 mM). The model solution samples were prepared as follows: At pH 9.5, 500 µL stock solution, 130 µL Milli-Q water (containing 2.69 mM TMSP-*d*_4_), and 70 µL D_2_O, pH adjusted by addition of sodium hydroxide solution; at pH 7.5, 500 µL stock solution, 130 µL phosphate buffer solution (see description below) and 70 µL of D_2_O.

#### 3.3.2. CSF Samples

CSF samples (*n* = 23) and two samples obtained by pooling remainders of those 23 unique samples were prepared for NMR analysis. CSF aliquots were allowed to thaw at room temperature (294 K), typically requiring about 5 min. The thawed aliquot was mixed by repeated inversion. 500 µL of CSF were transferred to a 1.5 mL standard Eppendorf tube and phosphate buffer solution (130 µL, 270 mM, pH 7.19) prepared from a mixture of K_2_HPO_4_ and NaH_2_PO_4_ in Milli-Q water, containing 0.11% (*w*/*v*) NaN_3_ and 2.69 mM deuterated sodium 3-trimethylsilylpropionate (TSP-*d*_4_) were added along with 70 µL of D_2_O. Upon sample mixing by repeated inversion, the mixture (700 µL) was transferred into a 5 mm NMR tube. Final phosphate concentration in each sample was 50 mM, and final TSP-*d*_4_ reference concentration was 0.5 mM. The pH of a prepared sample was determined prior and after data acquisition with a glass electrode to monitor the performance of the proposed buffer composition. Uncorrected pH values are reported. Samples were kept at 277 K while queued for acquisition.

### 3.4. NMR Spectroscopy

All ^1^H NMR spectra were collected on a 600 MHz Avance II+ spectrometer (Bruker BioSpin, Karlsruhe, Germany) equipped with a 5 mm TBI z-gradient pulsed-field gradient (PFG) room-temperature probe. One-dimensional ^1^H NMR spectra were acquired at 298 K using the first increment of a gradient enhanced nuclear Overhauser effect spectroscopy (NOESY) pulse sequence (noesypresat) with presaturation during a relaxation delay of 4 s and a mixing time of 10 ms for efficient water suppression. A total of 128 scans with a spectral width of 12,335 or 9590 Hz and 64k data points were recorded. Prior Fourier transformation, FID was multiplied by an exponential window function with a line-broadening factor of 0.5 Hz (if not stated otherwise), phased using zero-order terms (where possible), and referenced to the internal standard TSP-*d*_4_ (δ = 0.0 ppm). All NMR spectra were visually inspected for artifacts.

### 3.5. Chemometric Analysis

#### 3.5.1. Metabolic Fingerprinting and Profiling

One-dimensional proton spectra were baseline corrected, and 38 metabolites profiled using the database stored in Chenomx NMR suite 8.4 (Chenomx Inc., Edmonton, AB, Canada, 2018). For PLS regression, full resolution spectra in the spectral range of 0.5–10 ppm were used. The region containing the residual water signal and urea (4.7–6.4 ppm), in addition to the regions containing resonances of known contaminant signals (i.e., ethanol/isopropanol, 1.11–1.24 ppm and 3.63–3.695 ppm) were excluded. Spectra were baseline corrected and normalized to the total intensity.

#### 3.5.2. Statistical Analysis

Data matrix was imported into the R software. Statistical correlation analysis (STOCSY) [58] was used to aid the identification of ambiguous metabolites. STOCSY and PLS analysis were performed using “R” (cran.r-project.org), release 3.6.1 for Mac.

## 4. Conclusions

A simple and efficient protocol for the preparation of CSF NMR samples was devised allowing high throughput analysis and general applicability. Our investigation highlights the need for efficient pH control, and the suggested approach (i.e., employing a buffer), eliminates the necessity to adjust and check pH values of each individual sample.

Overall, our findings suggest that following sample preparation according to the proposed protocol, maintaining a biological pH and keeping samples refrigerated while queued for acquisition, allows even sensitive and low concentrated metabolites to remain stable for up to 48 h. In addition, we presented a detailed experimental description of CSF sample degradation using an NMR-based chemometric approach. Our observations support previous findings of changes in the levels of acetate, pyruvate and ascorbic acid associated with the duration of storage and we have quantified these (and other) changes. Previously documented observations [59], mainly those stating general sample instability, cannot be supported by our data when the samples are prepared according to the proposed protocol.

## Figures and Tables

**Table 1:** Pre-analytical factors and common steps in NMR sample preparation for metabolomic analysis of cerebrospinal fluid (CSF) described in the literature) [4,5,6,7,8,13,18,19,20,21,22,23,24,25,26,27,28,29,30,31,32,33,34,35,36,37,38,39].

Pre-Analytical Factor	Preparation Step	Conditions
Sample Volume	Variations of native sample volume. Sometimes native samples are lyophilized [22,31,36,42] and reconstituted and/or diluted [22,31,36,42,43,44].	Available native CSF volume ranges from 40–570 µL. Depending on experimental setup/objective, samples may be reconstituted/diluted with H_2_O/D_2_O.
Ionic Strength	Ionic strength buffering is rarely addressed [5,6,21,38].	Osmotic properties of buffer solutions vary (combinations of sodium/potassium phosphates/chlorides at varying concentrations)
Sample pH	Either adjusted with buffer solution [5,6,13,18,20,21,25,27,31,33,34,38,39], by adding HCl/NaOH solution [4,7,19,28,29,35,37], or ignored [8,23,24,26,32,36].	Adjusted sample pH range from 2.5–10. Buffer solutions with sodium/potassium phosphate concentration in the range of 35–166 mM.
Sample Solution Homogeneity	Mixing, centrifugation [4,6,7,26,38] and filtration [19,28,29,37] in order to homogenize samples and remove high MW compounds and cellular material.	Vortexing, mixing (e.g., by inversion), centrifugation, filtering, and deproteinization by precipitation are found.
Chemical Shift Reference and Internal Standard Lock Signal	TSP-*d*_4_ or DSS(-*d*_6_). Samples contain between 10% and 100% D_2_O as lock standard.	Typical concentration of reference compound is 0.5–1.25 mM in the final sample. Deuterated solvent is either used for reconstitution, as part of buffer solution, or added separately.
Storage and Stability	After preparation, while queued for acquisition, samples are stored under suitable conditions to maintain sample integrity.	Samples were either frozen (253 K) [45], typically stored in a cooled (277–279 K) environment [37,38,46], or sometimes kept at ambient temperature (294 K) [47].

**Table 2:** List of metabolites and concentrations (µM) determined in one of the pooled CSF samples (i.e., LT = low temperature pooled sample, see text).

Entry	Compound	Code	Concentration (µM)
1	2-Hydroxybutyrate	aHBA	18.4 ± 0.9
2	2-Hydroxyisovalerate	bHMB	4.4 ± 0.2
3	Acetate	AA	34.3 ± 1.7
4	Acetoacetate	AcA	4.3 ± 0.2
5	Acetone	Ac	7.4 ± 0.4
6	Alanine	Ala	25.2 ± 1.3
7	Ascorbate	AacA	109.9 ± 5.5
8	Caffeine	TMX	9.7 ± 0.5
9	Choline	CHO	2.3 ± 0.1
10	Citrate	Cit	90.9 ± 0.4
11	Creatine	Cr	27 ± 1.4
12	Creatinine	Cre	55.1 ± 2.8
13	Dimethylamine	DMA	1.5 ± 0.1
14	Dimethyl sulfone	DMS	7.2 ± 0.4
15	Ethanol	EtOH	1934.8 ± 96.7
16	Formate	FA	19.3 ± 1.0
17	Fructose	Frc	130.2 ± 6.5
18	Glucose	Glc	2013.3 ± 100.7
19	Glutamine	Gln	246.0 ± 12.3
20	Glycine	Gly	5.5 ± 0.3
21	Histidine	His	8.2 ± 0.4
22	Hypoxanthine	HX	3.7 ± 0.2
23	Isoleucine	Ile	3.5 ± 0.2
24	Isopropanol	iPrOH	224.1 ± 11.2
25	Lactate	Lac	907.7 ± 45.4
26	Leucine	Leu	7.8 ± 0.4
27	Lysine	Lys	16.2 ± 0.8
28	Mannose	Man	28.6 ± 1.4
29	Methanol	MeOH	45.4 ± 2.3
30	myo-Inositol	MIOL	66.3 ± 3.3
31	Phenylalanine	Phe	5.7 ± 0.3
32	Pyroglutamate	pyGlu	12.8 ± 0.6
33	Pyruvate	Pyr	12.8 ± 0.6
34	Threonine	Thr	31.0 ± 1.6
35	Trimethylamine N-oxide	TMAO	1.2 ± 0.1
36	Tyrosine	Tyr	8.2 ± 0.4
37	Valine	Val	11.1 ± 0.6
38	Xanthine	XAN	4.5 ± 0.2

## Supplementary Materials

The following are available online at https://www.mdpi.com/2218-1989/10/6/251/s1: Table S1: List of metabolites and concentrations [µM] determined in the pooled CSF sample ‘LT’ at the specified times of storage, Table S2: List of metabolites and concentrations [µM] determined in the pooled CSF sample ‘RT’ at the specified times of storage, Table S3: Relative concentration changes for pyruvate (PYR), and acetate (AA), with r^2^ ≈ 1 for different storage conditions in the pooled samples, Table S4: Relative metabolite concentrations of acetate and pyruvate determined via peak integration in 12 representative samples, Figure S1: ^1^H NMR spectrum showing all resonances and composition of the prepared model solution, Figure S2: Aromatic region of two ^1^H NMR spectra of the same CSF sample acquired with and without imidazole as an internal pH reference, Figure S3: Comparison of relative ethanol and isopropanol concentrations in replicated samples of four subjects, Figure S4: Supporting data for the assignment of caffeine in the analyzed CSF samples, Figure S5: Stacked plot of five ^1^H NMR spectra of sample ‘LT’ measured at the specified times after preparation, Figure S6: 1D STOCSY plot of spectral data (‘RT’ sample), Figure S7: Normality distribution and significance testing of acetate and pyruvate metabolite levels in 12 unique samples.
