# Supplementary material for: Pursuing Experimental Reproducibility: An Efficient Protocol for the Preparation of Cerebrospinal Fluid Samples for NMR-Based Metabolomics and Analysis of Sample Degradation
Source: Metabolites. 2020 Jun 16;10(6):251. doi: 10.3390/metabo10060251 (PMC7345835; doi:10.3390/metabo10060251)
Supplement: Supplementary file 1 [file metabolites-10-00251-s001.pdf]

Supplementary Materials for

Pursuing experimental reproducibility: an efficient protocol for the preparation of cerebrospinal fluid samples for NMR-based metabolomics and analysis of sample degradation

Benjamin Albrecht [1], Elena Voronina [1], Carola Schipke [2], Oliver Peters [3], Maria Kristina Parr [4], M. Dolores Díaz-Hernández [1,*] and Nils E. Schlörer [1,*]

[1] Department of Chemistry, Universität zu Köln, Greinstr.4, 50939 Köln, Germany; e-mail@e-mail.com
[2] Charité– Universitätsmedizin Berlin, corporate member of Freie Universität Berlin, Humboldt-Universität zu Berlin, & Berlin Institute of Health, Experimental & Clinical Research Center (ECRC), Lindenberger Weg 80, 13125 Berlin, Germany; schipke@predemtecdx.com
[3] Department of Psychiatry and Psychotherapy, Charité-Universitätsmedizin Berlin, Campus Benjamin Franklin, Hindenburgdamm 30, 12203 Berlin, Germany; oliver.peters@charite.de
[4] Institute of Pharmacy, Freie Universität Berlin, Königin-Luise-Str. 2+4, 14195 Berlin, Germany; maria.parr@fu-berlin.de
* Correspondence: mdiazher@uni-koeln.de (M.D.D.H.); nils.schloerer@uni-koeln.de (N.E.S.); Tel.: +49-221-470-3081 (N.E.S.)

Contents:

The four tables and seven figures in this supplementary file provide additional information in support of the results presented in the manuscript. Compound abbreviations are listed in the Table 2 of the main text.

In **Tables S1-S2** a list of metabolites and relative concentrations [mM] determined in both pooled CSF samples 'LT' and 'RT' (i.e. storage at low and room temperature, respectively) at the specified times of storage are presented. While in **Table S3** the relative concentration changes for pyruvate (PYR), and acetate (AA), for different storage conditions in the pooled samples are shown, the relative metabolite concentrations of acetate and pyruvate determined via peak integration in 12 representative samples belonging to one group of test subjects at the time of preparation (t = 0) and after storing the samples at 279 K for 1 month (t = 1 mo) are presented in **Table S4**.

Figure S1 is a ^{1}H NMR (600 MHz) spectrum showing all resonances and composition of the prepared model solution. In **Figure S2** the aromatic region of two ^{1}H NMR spectra of the same unique CSF sample acquired with (top) and without (bottom) imidazole as an internal pH reference (600 MHz, 298 K, phosphate buffer, pH 7.2, 10% D_2O) is shown. **Figure S3** displays in a graphic manner the comparison of relative ethanol (EtOH) and isopropanol (^{i}PrOH) concentrations in replicated samples (duplicated or triplicated) of four subjects (i.e. A, B, C and D). As is presented in **Figure S4**, identification of caffeine in the analyzed CSF samples (MSI level 2) could be reached by spectral profiling of a ^{1}H NMR spectrum of a CSF sample (black) performed in Chenomx using the internal 600 MHz metabolite library matching resonances of caffeine (red), assigning 3 of 4 characteristic resonances of caffeine. In **Figure S4** the 1D STOCSY plot generated from the data shows high correlation among three of the four characteristic resonances. The fourth resonance is likely masked by higher variance observed in the levels of glucose. To visualize the timely degradation of the sample stored at low temperature ('LT' 277 K), in **Figure S5** a stack plot of five ^{1}H NMR spectra measured at the specified times after preparation (600 MHz, 298 K, phosphate buffer 50 mM, pH 7.30-7.56, 10% D_2O, t (h)= 0, 2.5, 5, 25, 48) is displayed. Spectral regions (A) 8.50-7.05 ppm (intensity increased by a factor of 16), and (B) 4.73-1.85 ppm. XAN/TMX = xanthine/caffeine (tentative assignments). In **Figure S6** the 1D STOCSY plot of spectral data ('RT' sample) with acetate (d = 1.937 ppm), as driver showing high correlations with pyruvate ,($r^2 \approx 1$) and ascorbic acid ($r^2 \approx 0.8$) is presented. For the 12 samples presented in Table S4, acetate and pyruvate metabolite levels were examined for normal distribution using the Shapiro-Wilk test and the results are presented in **Figure S7**. Storage of samples at 279 K for a period of 1 months was associated with a significant decrease in pyruvate (T = 5.27, df = 11, p = 0.0003) and a significant increase in acetate (T = – 3.63, df = 11, p = 0.0040).

Table S1. List of metabolites and concentrations (μM) determined in the pooled CSF sample 'LT' (i.e. storage at low temperature) at the specified times of storage.

ID	COMPOUND		LT<br>0 h	LT<br>25 h	LT<br>48 h	LT<br>96 h
1	2-Hydroxybutyrate	aHBA	18.4±0.9	18.7±0.9	19±1	19.1±1
2	2-Hydroxyisovalerate	bHMB	4.4±0.2	3.6±0.2	4.1±0.2	4.1±0.2
3	Acetate	AA	34.3±1.7	34.2±1.7	35.6±1.8	39.9±2
4	Acetoacetate	AcA	4.3±0.2	5.6±0.3	4.9±0.2	4.4±0.2
5	Acetone	Ac	7.4±0.4	7.6±0.4	7.4±0.4	8.1±0.4
6	Alanine	Ala	25.2±1.3	24±1.2	24.4±1.2	24.9±1.2
7	Ascorbate	AscA	109.9±5.5	107.9±5.4	101±5.1	66.6±3.3
8	Caffeine	TMX	9.7±0.5	9.6±0.5	9.2±0.5	7.5±0.4
9	Choline	CHO	2.3±0.1	2.3±0.1	2±0.1	1.8±0.1
10	Citrate	CIT	90.9±4.5	87.8±4.4	92.4±4.6	88.6±4.4
11	Creatine	Cr	27±1.4	26.2±1.3	25.9±1.3	26.2±1.3
12	Creatinine	Cre	55.1±2.8	50.5±2.5	54.9±2.7	55.9±2.8
13	Dimethylamine	DMA	1.5±0.1	1.6±0.1	1.9±0.1	1.8±0.1
14	Dimethyl sulfone	DMS	7.2±0.4	7.4±0.4	6.9±0.3	7.6±0.4
15	Ethanol	EtOH	1934.8±96.7	2020.2±101	2047.9±102.4	2036.2±101.8
16	Formate	FA	19.3±1	20.5±1	20.3±1	22.5±1.1
17	Fructose	Frc	130.2±6.5	127.1±6.4	133.8±6.7	135±6.8
18	Glucose	Glc	2013.3±100.7	2017.4±100.9	2028.5±101.4	2007.2±100.4
19	Glutamine	Gln	246±12.3	271.9±13.6	265.5±13.3	260±13
20	Glycine	Gly	5.5±0.3	5.7±0.3	6.6±0.3	6.7±0.3
21	Histidine	His	8.2±0.4	5.6±0.3	6.4±0.3	8.8±0.4
22	Hypoxanthine	HX	3.7±0.2	3.3±0.2	3.1±0.2	3.7±0.2
23	Isoleucine	Ile	3.5±0.2	3.9±0.2	4.2±0.2	3.7±0.2
24	Isopropanol	iPrOH	224.1±11.2	225.4±11.3	227.4±11.4	248.9±12.4
25	Lactate	Lac	907.7±45.4	910.5±45.5	878.4±43.9	906.2±45.3
26	Leucine	Leu	7.8±0.4	6.7±0.3	6.9±0.3	6.8±0.3
27	Lysine	Lys	16.2±0.8	14.6±0.7	14.2±0.7	16±0.8
28	Mannose	Man	28.6±1.4	29.4±1.5	29.9±1.5	29.8±1.5
29	Methanol	MeOH	45.4±2.3	44.8±2.2	44.1±2.2	43.9±2.2
30	myo-Inositol	MIOL	66.3±3.3	61.2±3.1	61.7±3.1	59.9±3
31	Phenylalanine	Phe	5.7±0.3	6.5±0.3	6.2±0.3	6.8±0.3
32	Pyroglutamate	PyGlu	12.8±0.6	12.5±0.6	14.9±0.7	14.5±0.7
33	Pyruvate	Pyr	78.5±3.9	79.2±4	78.5±3.9	79.7±4
34	Threonine	Thr	31±1.6	34.8±1.7	36.5±1.8	34.2±1.7
35	Trimethylamine N-oxide	TMAO	1.2±0.1	1.7±0.1	1.6±0.1	1.7±0.1
36	Tyrosine	Tyr	8.2±0.4	9.5±0.5	9.5±0.5	11±0.6
37	Valine	Val	11.1±0.6	10.6±0.5	11±0.6	10.9±0.5
38	Xanthine	Xan	4.5±0.2	4.4±0.2	4.6±0.2	5.8±0.3

Table S2. List of metabolites and concentrations (μM) determined in the pooled CSF sample 'RT' (i.e. storage at room temperature) at the specified times of storage.

ID	COMPOUND		RT<br>0 h	RT<br>2 h	RT<br>20 h	RT<br>52 h	RT<br>100 h
1	2-Hydroxybutyrate	aHBA	21.5±1.1	19.5±1	21.4±1.1	21.6±1.1	21±1.1
2	2-Hydroxyisovalerate	bHMB	5.5±0.3	5.6±0.3	7.4±0.4	5.8±0.3	5.5±0.3
3	Acetate	AA	17±0.9	24.9±1.2	66.7±3.3	107.2±5.4	253.4±12.7
4	Acetoacetate	AcA	4.4±0.2	4.4±0.2	4.2±0.2	5.1±0.3	4.1±0.2
5	Acetone	Ac	5.8±0.3	6±0.3	5.8±0.3	6.2±0.3	6±0.3
6	Alanine	Ala	32.3±1.6	32.3±1.6	32.6±1.6	29.9±1.5	23.2±1.2
7	Ascorbate	AscA	145.8±7.3	141±7.1	71.8±3.6	0±0	0±0
8	Caffeine	TMX	13.6±0.7	13.1±0.7	12.1±0.6	12.6±0.6	14±0.7
9	Choline	CHO	2.4±0.1	2.3±0.1	2.4±0.1	2.4±0.1	1.5±0.1
10	Citrate	CIT	116.8±5.8	115.4±5.8	114.9±5.7	106±5.3	111.4±5.6
11	Creatine	Cr	40.4±2	40.7±2	39.9±2	40.6±2	39.2±2
12	Creatinine	Cre	57.4±2.9	57.4±2.9	56.3±2.8	58.3±2.9	54.8±2.7
13	Dimethylamine	DMA	3.9±0.2	4.7±0.2	6±0.3	6.7±0.3	7±0.4
14	Dimethyl sulfone	DMS	13.2±0.7	13.1±0.7	13.2±0.7	13.4±0.7	12.4±0.6
15	Ethanol	EtOH	3448.5±172.4	3437.2±171.9	3477.3±173.9	3490.6±174.5	3448.1±172.4
16	Formate	FA	34.2±1.7	34.2±1.7	32.3±1.6	33.4±1.7	33.2±1.7
17	Fructose	Frc	177.3±8.9	162.4±8.1	151.3±7.6	139.9±7	105.6±5.3
18	Glucose	Glc	2267±113.4	2270.6±113.5	2251±112.6	2271.9±113.6	2161.1±108.1
19	Glutamine	Gln	288.7±14.4	309±15.5	309.4±15.5	314.1±15.7	294.4±14.7
20	Glycine	Gly	8.5±0.4	8.5±0.4	7.8±0.4	7.4±0.4	5.5±0.3
21	Histidine	His	9.3±0.5	9.5±0.5	10.4±0.5	9.7±0.5	10.1±0.5
22	Hypoxanthine	HX	3.7±0.2	3.4±0.2	3.3±0.2	3.5±0.2	0±0
23	Isoleucine	Ile	5.2±0.3	5.9±0.3	6.6±0.3	6.1±0.3	2.5±0.1
24	Isopropanol	iPrOH	515.6±25.8	442.4±22.1	429.3±21.5	451±22.6	413.1±20.7
25	Lactate	Lac	1173.3±58.7	1135.1±56.8	1130.7±56.5	1149.9±57.5	1147.1±57.4
26	Leucine	Leu	8.9±0.4	10.1±0.5	8.4±0.4	9±0.5	6.5±0.3
27	Lysine	Lys	18.1±0.9	18.9±0.9	19.5±1	19.4±1	12.8±0.6
28	Mannose	Man	33.2±1.7	31±1.6	40.1±2	32.8±1.6	32.6±1.6
29	Methanol	MeOH	33.5±1.7	31.6±1.6	32.9±1.6	31.7±1.6	31.6±1.6
30	myo-Inositol	MIOL	116±5.8	114.8±5.7	109.8±5.5	102.9±5.1	112.4±5.6
31	Phenylalanine	Phe	8.9±0.4	8.2±0.4	7.9±0.4	9±0.5	7.5±0.4
32	Pyroglutamate	PyGlu	11.7±0.6	14.7±0.7	17.5±0.9	24.9±1.2	30.5±1.5
33	Pyruvate	Pyr	92.1±4.6	90.1±4.5	49.6±2.5	13.1±0.7	9.1±0.5
34	Threonine	Thr	35.7±1.8	35.7±1.8	33.1±1.7	35.6±1.8	19.2±1
35	Trimethylamine N-oxide	TMAO	0.9±0.1	1.1±0.1	1.2±0.1	1.1±0.1	1.2±0.1
36	Tyrosine	Tyr	10.1±0.5	11.2±0.6	10.1±0.5	10.7±0.5	9±0.5
37	Valine	Val	13.9±0.7	14.3±0.7	15.3±0.8	15.3±0.8	11±0.6
38	Xanthine	Xan	21.5±1.1	19.5±1	21.4±1.1	21.6±1.1	21±1.1

Table S3. Relative concentration changes for pyruvate (PYR), and acetate (AA), with $r^2 \approx 1$ for different storage conditions in the pooled samples.

		**Δc (μM)**	
	**Δ*t***	**PYR**	**AA**
**RT**	20 h	–42.5±2.1	49.7±2.5
**LT**	21 d	–40.8±2.0	48.4±2.4

Table S4. Metabolite concentrations (mM) of acetate and pyruvate determined via peak integration in 12 representative samples belonging to one group of test subjects at the time of preparation (t = 0) and after storing the samples at 279 K for 1 month (t = 1 mo).

	Acetate		Pyruvate	
	t = 0	*t* = 1 mo	*t* = 0	*t* = 1 mo
**Sample 1**	0.0339±0.0017	0.033±0.0017	0.0057±0.0003	0
**Sample 2**	0.039±0.002	0.039±0.002	0	0
**Sample 3**	0.0356±0.0018	0.042±0.0021	0.0092±0.0005	0.003±0.0002
**Sample 4**	0.0252±0.0013	0.0471±0.0024	0.0176±0.0009	0.0036±0.0002
**Sample 5**	0.0303±0.0015	0.0323±0.0016	0.009±0.0005	0.0041±0.0002
**Sample 6**	0.0399±0.002	0.0623±0.0031	0.0117±0.0006	0.0044±0.0002
**Sample 7**	0.0366±0.0018	0.0407±0.002	0.0104±0.0005	0.0044±0.0002
**Sample 8**	0.0348±0.0017	0.0399±0.002	0.0024±0.0001	0
**Sample 9**	0.0362±0.0018	0.0387±0.0019	0.0134±0.0007	0.0038±0.0002
**Sample 10**	0.0144±0.0007	0.0266±0.0013	0.0119±0.0006	0.0021±0.0001
**Sample 11**	0.0192±0.001	0.0387±0.0019	0.0203±0.001	0.0015±0.0001
**Sample 12**	0.0276±0.0014	0.0387±0.0019	0.0207±0.001	0.005±0.0003

Figure S1. 1D ^{1}H NMR (600 MHz, 298 K, phosphate buffer, pH 7.5, 10% D_2O) spectrum showing all resonances and composition of the prepared model solution. **Note:** For compound abbreviations, see **Table 2** main text.

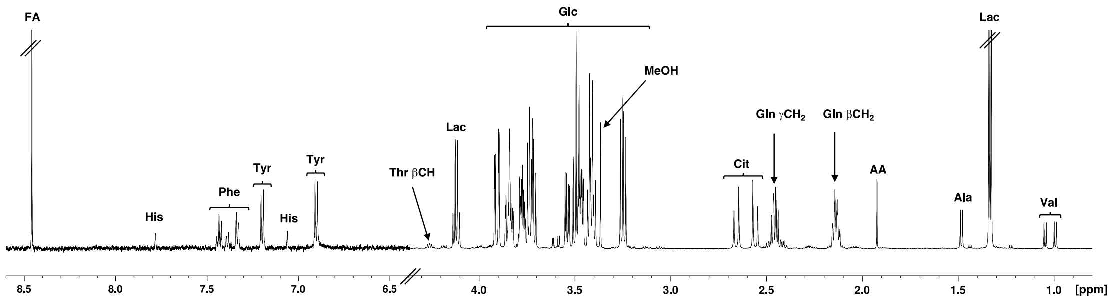

Figure S2. (Top-down) Aromatic region of two ^1H NMR spectra of the same unique CSF sample acquired with and without imidazole as an internal pH reference (600 MHz, 298 K, phosphate buffer, pH 7.2, 10% D_2O)

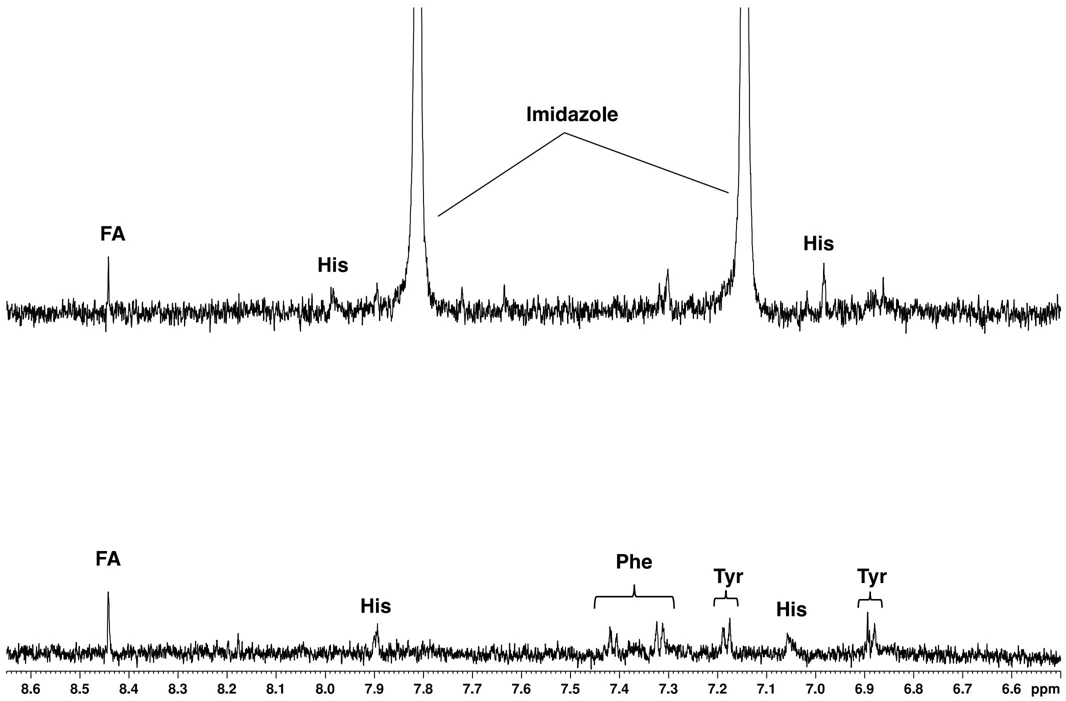

Figure S3. Comparison of relative ethanol (EtOH) and isopropanol (^{i}PrOH) concentrations in replicated samples (duplicated or triplicated) of four subjects (i.e. A, B, C and D). Similar ratios of c_{EtOH}/c_{iPrOH} were found. A mean ratio of 12.63 with a standard deviation of 4.03 was determined. The occurrence of similar ratios can be easily explained by the fixed composition of these two commonly used agents in skin disinfectant applied prior to the sampling procedure. Different concentrations of ethanol and isopropanol amongst duplicates and triplicates of the same patient can be explained by the order of aliquoting samples (not necessarily represented by the numbering of samples).

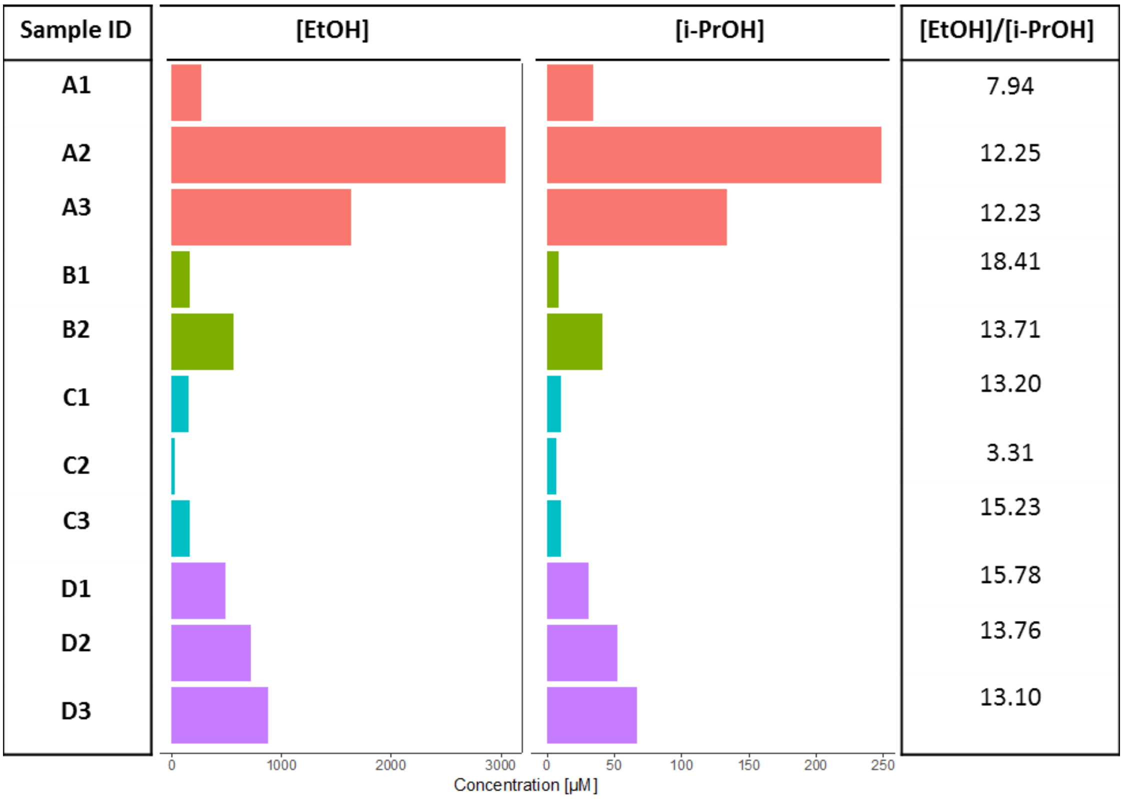

Figure S4. Supporting data for the assignment of caffeine in the analyzed CSF samples. MSI level 2 could be reached by spectral profiling, assigning 3 of 4 characteristic resonances, as well as a STOCSY analysis. (A) Spectral profiling of ^1H NMR spectrum of a CSF sample (black) performed in Chenomx using the internal 600 MHz metabolite library matching resonances of caffeine (red). (B) 1D STOCSY plot generated from the data acquired from 23 unique CSF samples showing high correlation among three of the four characteristic resonances. The fourth resonance is likely masked by greater variance observed in the levels of glucose.

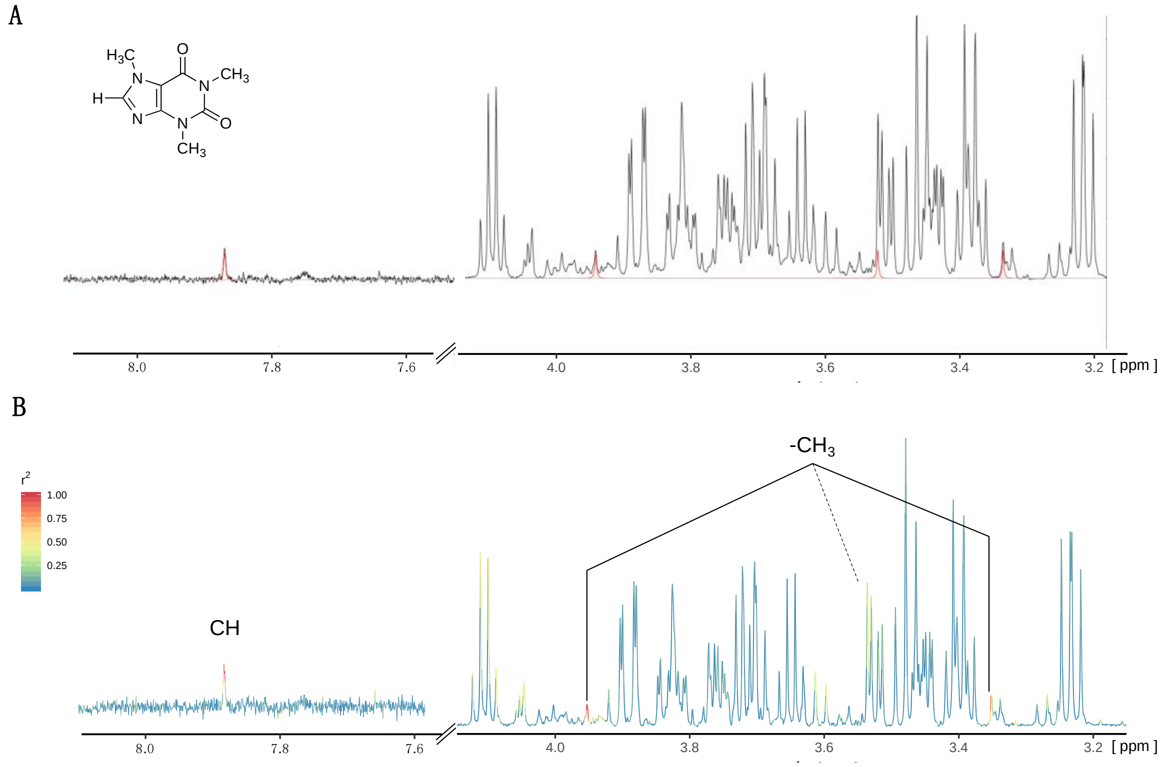

Figure S5. Stack plot of five 1H NMR spectra of sample 'LT' measured at the specified times after preparation (600 MHz, 298 K, phosphate buffer 50 mM, pH 7.30-7.56, 10% D2O, t (h)= 0, 2.5, 5, 25, 48). The sample was stored at 277 K between measurements. Spectral regions (A) 8.50-7.05 ppm (intensity increased by a factor of 16), and (B) 4.73-1.85 ppm. XAN/TMX = xanthine/caffeine (tentative assignments),

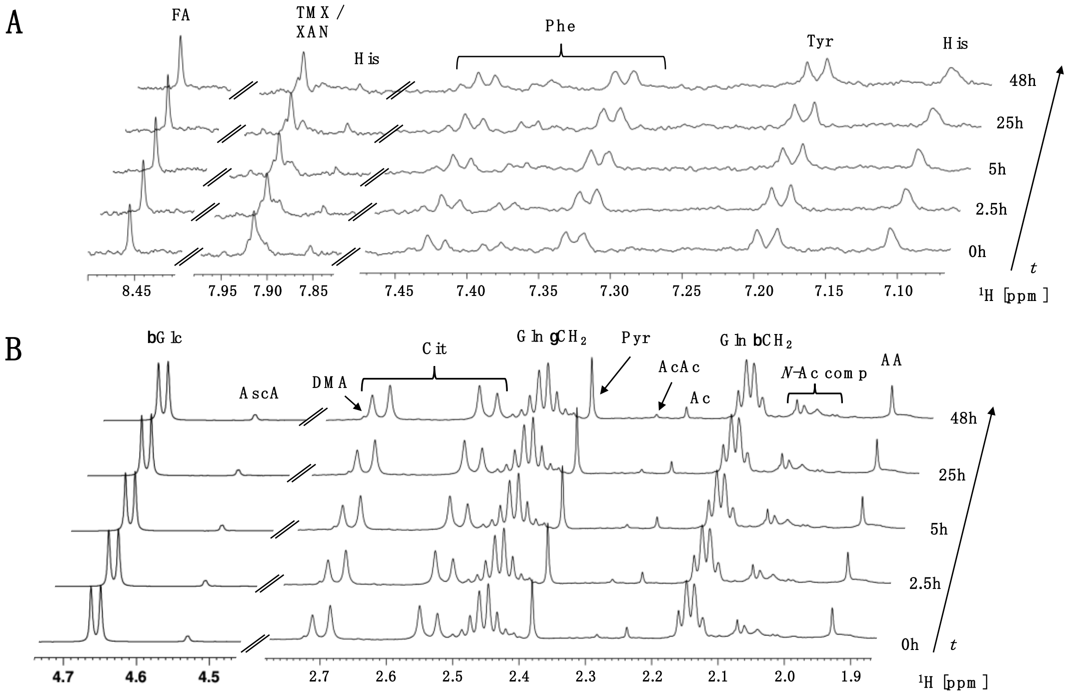

Figure S6. 1D STOCSY plot of spectral data ('RT' sample) with acetate (AA, δ = 1.937 ppm), as driver showing high correlations, $r^2 \approx 1$ with pyruvate (Pyr), and $r^2 \approx 0.8$ with ascorbic acid (AscA).

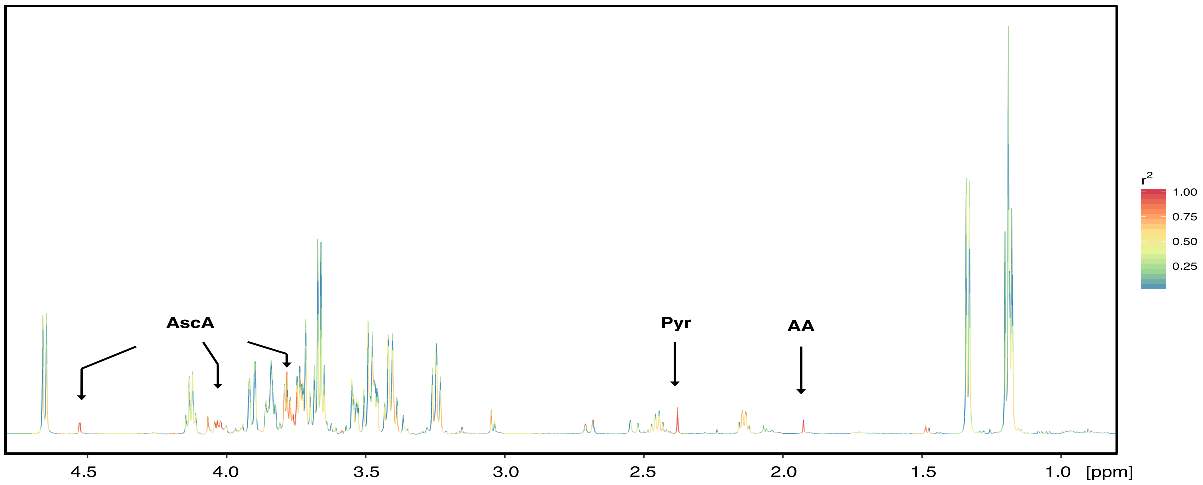

Figure S7. Acetate and pyruvate metabolite levels in the 12 samples were examined for normal distribution using the Shapiro-Wilk test. Paired samples t-tests were performed to compare metabolite levels at the time of sample preparation ($t = 0$) and after a period of storage/degradation ($t = 1$ mo). Storage of samples at 279 K for a period of 1 months was associated with a significant decrease in pyruvate (T = 5.27, df = 11, $p = 0.0003$) and a significant increase in acetate (T = –3.63, df = 11, $p = 0.0040$).

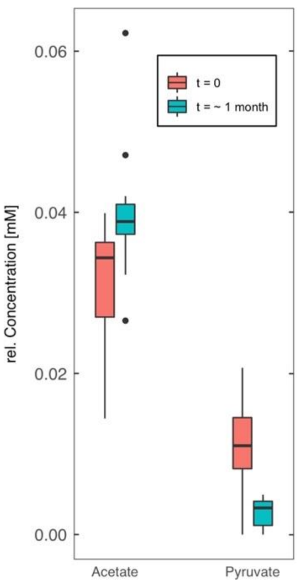

	***t* = 0**	***t* = 1 mo**
**Acetate**	0.0310 ± 0.0023	0.0399 ± 0.0025
**Pyruvate**	0.0110 ± 0.0019	0.0026 ± 0.0005
